# Cardiovascular Insufficiency, Abdominal Sepsis, and Patients' Age Are Associated with Decreased Paraoxonase-1 (PON1) Activity in Critically Ill Patients with Multiple Organ Dysfunction Syndrome (MODS)

**DOI:** 10.1155/2019/1314623

**Published:** 2019-02-11

**Authors:** Iwona Bednarz-Misa, Magdalena Mierzchala-Pasierb, Patrycja Lesnik, Sylwia Placzkowska, Krzysztof Kedzior, Andrzej Gamian, Malgorzata Krzystek-Korpacka

**Affiliations:** ^1^Department of Medical Biochemistry, Wroclaw Medical University, Wroclaw, Poland; ^2^Department of Anesthesiology and Intensive Therapy of Wroclaw Medical University, Wroclaw, Poland; ^3^Department of Professional Training in Clinical Chemistry, Wroclaw Medical University, Wroclaw, Poland; ^4^Institute of Immunology and Experimental Therapy, Polish Academy of Sciences, Wroclaw, Poland

## Abstract

Oxidative stress and uncontrolled inflammation are hallmarks of sepsis, leading to organ failure and death. As demonstrated in animal studies, oxidative stress can be alleviated by antioxidant therapies. Paraoxonase-1 (PON1) is a serum-based antioxidant, anti-inflammatory agent, detoxifier, and quorum-sensing factor found to be a prognostic marker in sepsis. However, its associations with multiple organ dysfunction syndrome (MODS), a complication of sepsis and the leading cause of death in the surgical intensive care units (ICU), as well as with specific organ dysfunction, infection site, and invading pathogen remain unknown. Therefore, we measured arylesterase activity of PON1 in 87 individuals (35 with MODS) and related it to the clinical type, organ failure, infection site, pathogens, and hematological and biochemical indices of inflammation at admission to ICU and during a five-day follow-up. Suitability of PON1 and its indices derived from a follow-up as biomarkers in MODS was evaluated as well. MODS was associated with decreased PON1, more so in patients with septic shock, displaying an excellent accuracy as a marker of MODS (91%) and a fair one as a marker in differentiating septic shock from severe sepsis (76%). Decreased admission PON1 accompanied cardiovascular insufficiency (CVI), and, as its marker, PON1 displayed a good accuracy (82%). It was also associated with the abdomen as a site of infection but not with an invading pathogen. In multivariate analysis, 50% of variability in PON1 activity in patients with MODS was explained by the patients' age, CVI, and abdomen as a site of infection. Patients with septic shock, CVI, and abdominal MODS had distinctly different dynamics of PON1 during a follow-up. Mean PON1 activity during the follow-up reflected the associations observed for admission PON1 but was also significantly associated with metabolic dysfunction. Our results show PON1 potential as a biomarker in MODS, particularly as an indicator of CVI.

## 1. Introduction

Multiple organ dysfunction syndrome (MODS) is a complication of severe sepsis and septic shock and a leading cause of death in the surgical intensive care units (ICU). MODS development increases the mortality among ICU patients by 20-fold. Mortality is proportional to the number of damaged organs as well as the duration of organ dysfunction. The prognosis in MODS is worsened by advanced age and preexisting serious illness. Since the incidence of sepsis is rising in parallel to the ageing of societies, an increase in the number of elderly septic patients with comorbidities, being at a greater risk for MODS, might be expected [1]. Early diagnosis and effective treatment are crucial for preventing MODS and surviving sepsis; both, however, are hindered by great heterogeneity of the condition [2].

Oxidative stress and uncontrolled inflammation are common denominators in sepsis. Oxidative imbalance causes mitochondrial dysfunction leading to cellular energetic failure and, through overproduction of NO^•^, to the general vasodilatation accompanied by poor responsiveness to vasoconstrictor agents (reviewed in [3]). It also promotes and mediates systemic inflammatory response syndrome (SIRS). In turn, uncontrolled inflammation, a hallmark of severe sepsis, leads to organ failure and, in up to 60% of patients [4], to death. Experimental antioxidant therapies have been successful in alleviation of oxidative imbalance in animal models of sepsis [3].

Paraoxonase-1 (PON1) (EC 3.1.8.1) is an extracellular esterase with broad substrate specificity, a serum-based antioxidant involved in the defense against lipid peroxidation [5], and an anti-inflammatory response [6]. Recently, the intestine has been demonstrated to be an important source of circulating enzymes in addition to the liver [7]. Moreover, the PON family of enzymes has been shown to utilize a variety of lactones as their primary substrates [8]. Among them are acyl homoserine lactones [6] and quorum-sensing mediators of Gram-negative bacteria [9]. Accordingly, a role of the quorum-quenching factor has been suggested for PON1 [7] and PON2 [10]. The clinical significance of PON1 has been evaluated mainly in cardiovascular diseases (reviewed in [11]). However, diminished enzyme activity has also been observed in infectious diseases (reviewed in [12]) as well as both acute and low-grade inflammatory conditions [13–15]. Despite potential relevance of PON1 for sepsis development and progression, data on the enzyme status in sepsis remains scanty. Nonetheless, all studies concerning PON1 clearly indicate a significant drop in PON1 activity in adult [16–20] as well as pediatric [21] and neonatal sepsis [22].

Unlike in previous studies focused on survival, our objective was to determine PON1 activity in critically ill patients with MODS and to put it in the context of the clinical type, ongoing inflammation, invading pathogen, infection site, and specific organ failure. We also aimed to evaluate PON1 suitability as a biomarker in sepsis. Since clinical improvement is associated with attenuation of inflammation, the time course of biomarkers has been suggested to better reflect patients' outcome than single evaluation upon admission [23–25]. Therefore, we additionally examined various measures of changes in PON1 activity during a 5-day follow-up.

## 2. Materials and Methods

### 2.1. Patients

This study is a part of research on potential biomarkers of sepsis conducted in Wroclaw Medical University between November 2005 and February 2007 [25, 26]. Of the originally enrolled 40 patients meeting consensus criteria for severe sepsis and septic shock [27], 35 patients presenting with MODS were evaluated in this study. Severe sepsis was recognized in a presence of acute failure of at least one organ in addition to systemic inflammatory response syndrome (SIRS) (at least two of the following symptoms are met: temperature > 38°C or <36°C; heart rate > 90 beats/min.; respiratory rate > 20 breaths/min. or arterial CO_2_ < 32 mmHg; and WBC > 12 × 10^9^ cells/L or <4 × 10^9^ cells/L or presence of immature form > 10%) and to clinically or microbiologically confirmed infection. Septic shock was defined as a severe sepsis with systolic blood pressure < 90 mmHg for at least one hour despite adequate fluid resuscitation or as the necessity for vasopressive therapy [28]. The MODS was defined as a progressive yet reversible dysfunction of at least two organs [28]. The organ/system function was evaluated using Sepsis-related Organ Failure Assessment (SOFA) [29]. The cardiovascular insufficiency (CVI) was defined as systolic blood pressure < 90 mmHg or mean blood pressure ≤ 70 mmHg for at least one hour despite proper fluid administration and adequate hydration status or as the need to administer vasoconstrictors for at least one hour to maintain systolic blood pressure ≥ 90 mmHg or mean blood pressure > 70 mmHg. The kidney failure (KF) was defined as urine output < 0.5 mL/kg of body mass/hours for two hours despite proper fluid transfusion or as serum creatinine exceeding the upper limit of the normal range by twofold. The liver failure was defined as serum bilirubin concentration exceeding the upper limit of the normal range by threefold and as clinically manifested jaundice and INR > 3.0. The pulmonary dysfunction was defined as PaO_2_/FiO_2_ ≤ 250 mmHg (≤200 in patients with pneumonia or other pulmonary diseases) and pulmonary capillary wedge pressure < 18 mmHg (if measured), and the hematological dysfunction was defined as platelet count < 100 × 10^9^/L or as its drop by 50% as compared to the maximal value within the last three days. The metabolic dysfunction was defined as lactate > 2 mmol/L or arterial pH ≤ 7.3 or BE ≤−5.0 mEq/L. Additionally, the Acute Physiology and Chronic Health Evaluation II (APACHE II) [30] score was calculated to assess sepsis severity. All patients were treated according to the accepted standards for severe sepsis and septic shock (antibiotic therapy, mechanical ventilation, fluid resuscitation, and vasopressive therapy) and monitored until their discharge from the intensive care unit or death. Detailed characteristics of patients are presented in Table 1.

A group of 52 blood donors from the Regional Center of Blood Donation and Therapy, Wroclaw, Poland, served as healthy controls. Inclusion criteria were as follows: age > 40 yrs., no significant health history, no active inflammation, no pregnancy, and routinely evaluated laboratory parameters within the normal range.

There were no differences concerning age and gender distribution (females/males) between controls and septic patients (respectively 49.5 vs. 53.4 years, *p* = 0.300, and 28/24 vs. 18/17, *p* = 0.831).

### 2.2. Ethical Considerations

The study protocol was approved by the Medical Ethics Committee of Wroclaw Medical University, Wroclaw, Poland, and the study was conducted in accordance with the Helsinki Declaration of 1975, as revised in 1983, and an informed consent has been obtained from patients' relatives.

### 2.3. Analytical Methods

Blood drawn by venipuncture was collected into serum-separator tubes on admission (1st day). Additionally, if available, blood was sampled on the 2nd, 3rd, and 5th days. Serum was obtained from clotted (15 min., RT) and centrifuged (10 min., 720 × *g*) blood and stored at -80°C until analyses.

PON1 activity was measured in sera as a rate of phenyl acetate (Sigma-Aldrich, St. Louis, MO) hydrolysis (PON1 arylesterase activity), according to the Arylesterase/Paraoxonase Assay Kit protocol (ZeptoMetrix Co., Buffalo, NY) (intra-assay CV = 3.0%). One unit (U) of enzyme activity was defined as one mmol of phenol released at 25°C per liter of serum per minute. Arylesterase activity, not being affected by common PON1 polymorphisms, has been demonstrated to represent enzyme concentration [31].

Interleukin 6 (IL-6) was assessed using the immunoenzymatic method using respective ELISA (Milenia Biotec, Bad Nauheim, Germany). The endotoxin level (lipopolysaccharide, LPS) was assayed by using LAL Chromogenic Endpoint Assay (HyCult biotechnology, Uden, Netherlands). For the purpose of correlation analysis, data on procalcitonin (PCT) (assessed by the immunoluminometric method using LUMItest PCT from Brahms Diagnostica GmbH, Berlin, Germany) and lipopolysaccharide-binding protein (LBP) (measured immunoenzymatically with commercially available ELISA tests from HyCult Biotechnology, Uden, Netherlands) were retrieved from our previous work [25]. CRP and other laboratory indices were assessed with routine automatic procedures.

### 2.4. Statistical Analysis

Data distribution and equality of variances were tested using the Kolmogorov-Smirnov and Levene tests; log transformation was done when appropriate (IL-6, PCT, and creatinine). If not otherwise stated, data are presented as means (geometric means in case of log-transformed data) with 95% CI and analyzed using ANOVA and the *t*-test for independent samples with Welch correction if appropriate. Frequency analysis was conducted using the Fisher exact test and correlation analysis using the Pearson (continuous data) or Spearman (categorical data) correlation tests. Age was identified as a possible confounder and in the association studies was adjusted for using analysis of covariance (ANCOVA). Two-way ANOVA was used to coexamine the effect of categorical variables on PON1. Multiple regression analysis (stepwise method) was used to evaluate the strength of identified associations. Changes in PON1 activity (ΔPON1) as well as in other clinical and biochemical parameters were calculated as measurements on the 3rd day subtracted from measurements on the 1st day. Mean PON1 activities throughout the course of observation were calculated as well. Repeated measures ANOVA (2-factor study with repeated measures on one factor) was applied to evaluate the effect of categorical variables on the PON1 time course.

Receiver operating characteristic (ROC) curve analysis was applied to evaluate PON1 as a biomarker in sepsis. Overall performance of PON1, defined as overall marker accuracy, was expressed as the area under the ROC curve (AUC). Marker's sensitivities and specificities corresponding with the optimal cut-off value were calculated as well. The following interpretation of AUCs was used: 0.9-1 = excellent, 0.8-0.9 = good, 0.7-0.8 = fair, 0.6-0.7 = poor, and 0.5-0.6 = fail. Additionally, likelihood ratios (LRs), defined as “the ratio of the probability of a positive test result if the outcome is positive (true positive) to the probability of a positive test result if the outcome is negative (false positive)” for LR+ and “the ratio of the probability of a negative test result if the outcome is positive to the probability of a negative test result if the outcome is negative” for LR-, were calculated [32].

All tests were two-sided and *p* values ≤ 0.05 were considered significant. All statistical analyses were conducted using MedCalc Statistical Software version 18.2.1 (MedCalc Software bvba, Ostend, Belgium; http://www.medcalc.org; 2018).

## 3. Results

### 3.1. Admission PON1 and the Disease Severity and Outcome

**Figure 1:**
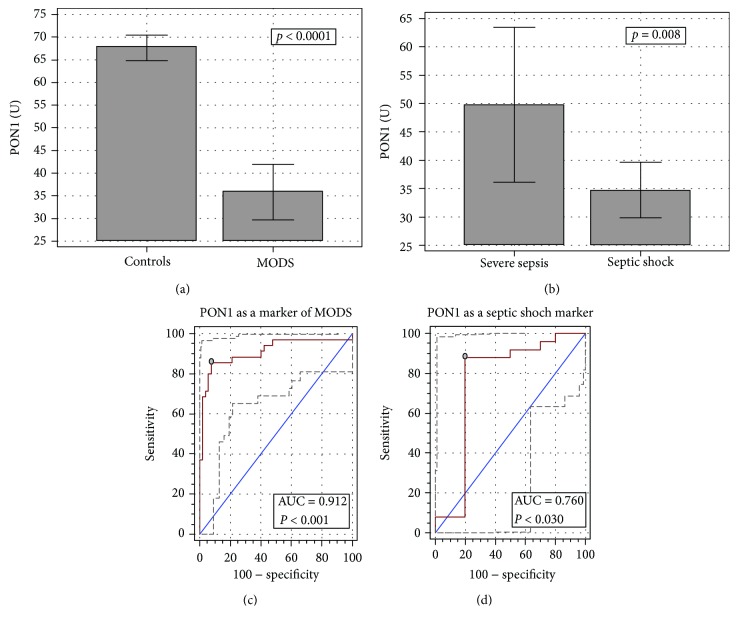
PON1 activities in healthy controls and MODS (a), the effect of the clinical type of MODS (b), and PON1 as a marker of MODS (c) and septic shock (d). Data on panels (a–b) presented as means with 95% CI and analyzed using the *t*-test for independent samples. Data on panels (c–d) presented as ROC curves with 95% CI. The diagonal line represents the chance marker with no discriminative power (AUC = 0.5), and a grey dot represents an optimal cut-off value. MODS: multiple organ dysfunction syndrome. (a) Significantly different from healthy controls. (b) Significantly different from severe sepsis. AUC: area under the receiver operating characteristic (ROC) curve.

PON1 activity on admission was lower in septic patients presenting with MODS than in healthy controls (Figure 1(a)). Among MODS patients, it was more pronouncedly decreased in septic shock patients (Figure 1(b)). PON1 activity remained lower in septic shock as compared to severe sepsis following adjustment to patients' age (*p* = 0.027). However, there was no correlation between PON1 and APACHE II and SOFA, either on admission or on any other day.

A drop in PON1 activity was an excellent indicator of MODS with overall accuracy of 91% and 85.7% sensitivity and 92.3% specificity corresponding with a cut-off at ≤51.6 U (Figure 1(c)). The likelihood ratio of the positive result (LR+) corresponding with this cut-off was 11.1 and that of a negative one (LR-) was 0.15.

PON1 was also a fair marker differentiating severe sepsis and septic shock with 76% accuracy and 88% sensitivity and 80% specificity corresponding with a cut-off at ≤42.3 U (Figure 1(d)). Corresponding LR+ and LR- were 4.4 and 0.15, respectively.

Decreases in the enzyme activity were not associated with the patients' outcome (40.3 U (33-48) in survivors and 39.2 U (32-46) in nonsurvivors, *p* = 0.828).

### 3.2. Admission PON1 Activity and Organ Dysfunction

**Figure 2:**
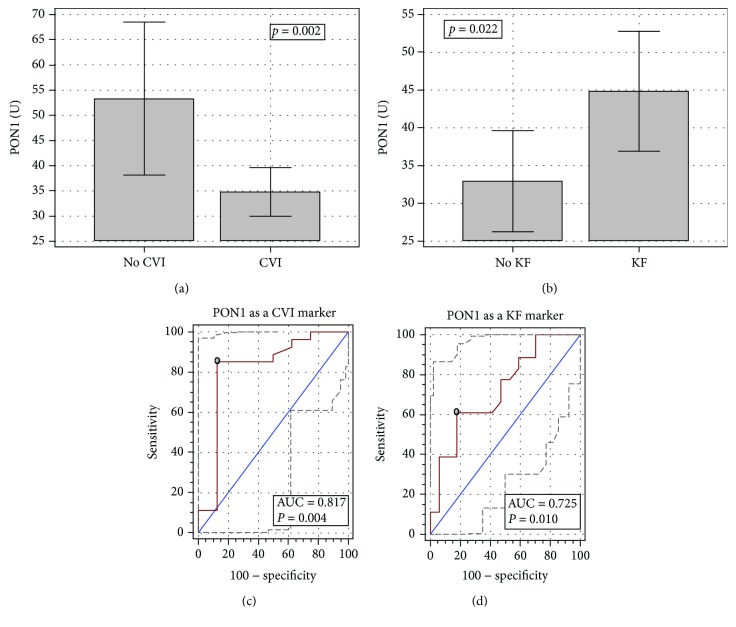
PON1 association with cardiovascular insufficiency (a) and kidney failure (b). PON1 as a marker of cardiovascular insufficiency (c) and kidney failure (d). Data on panels (a–b) presented as means with 95% CI and analyzed using the *t*-test for independent samples. Data on panels (c–d) presented as ROC curves with 95% CI. The diagonal line represents the chance marker with no discriminative power (AUC = 0.5), and a grey dot represents an optimal cut-off value. CVI: cardiovascular insufficiency; KF: kidney failure; AUC: area under the receiver operating characteristic (ROC) curve.

A significant drop in PON1 activity accompanied patients with cardiovascular insufficiency (CVI). In patients with kidney failure (KF), on the contrary, PON1 activity was higher (Figure 2(a) and 2(b)). When analyzed together, in the age-adjusted analysis, both CVI (*p* = 0.039) and KF (*p* = 0.010) remained significantly and independently from each other associated with PON1 activity. Still, as compared to healthy individuals, patients with KF had significantly lower PON1 activity (44.8 U (36.9-52.8) vs. 66.2 U (63.6-68.9), *p* < 0.0001).

Overall, the decreased PON1 activity was a good marker of CVI (Figure 2(c)). PON1 activity ≤ 42.3 U had 85.2% sensitivity and 87.5% specificity in discriminating CVI. Corresponding LR+ and LR- were 6.8 and 0.17, respectively. In turn, PON1 activity > 40.8 U had 61% sensitivity and 82.3% specificity in discriminating KF, and an overall performance of the enzyme as a KF marker was fair (Figure 2(d)). Corresponding LR+ and LR- were 3.5 and 0.47, respectively.

There was no significant difference in PON1 activity with respect to any other organ/system failure (*p* = 0.144 for metabolic, *p* = 0.554 for respiratory, *p* = 0.286 for hematological systems, and *p* = 0.798 for the liver).

### 3.3. Admission PON1 and the Infection Site and Invading Pathogen

**Figure 3:**
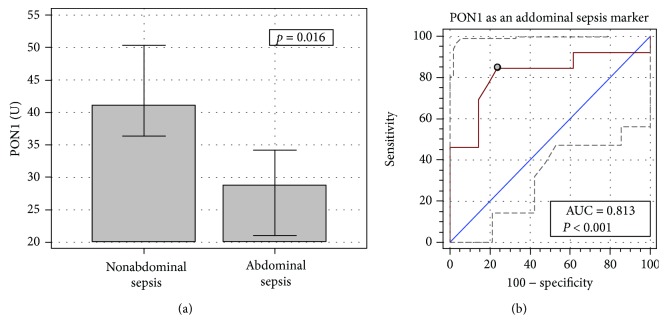
PON1 association with the abdomen as a site of infection (a) and the enzyme as a marker of abdominal sepsis (b). Data on panel (a) presented as means with 95% CI and analyzed using the *t*-test for independent samples. Data on panel (b) presented as ROC curves with 95% CI. The diagonal line represents the chance marker with no discriminative power (AUC = 0.5), and a grey dot represents an optimal cut-off value. AUC: area under the receiver operating characteristic (ROC) curve.

In patients stratified by the infection site, the most pronounced decreases in PON1 were observed in abdominal infections (28.2 U (24-33)), significantly lower than in the case of the pulmonary (45 U (38.5-51.5)), blood (44.3 U (34-54.5)), and other site (46.4 U) infections (*p* = 0.017). Abdominal infection was significantly associated with PON1 decreases also after adjusting for age (*p* = 0.004). Therefore, we analyzed abdominal MODS against all others (nonabdominal) and evaluated PON1 as a marker of abdominal MODS (Figure 3). A drop in PON1 activity was a good indicator of abdominal MODS with overall accuracy of 81% and 84.6% sensitivity and 76.2% specificity, corresponding with a cut-off at ≤31.5 U. Corresponding LR+ and LR- were 3.6 and 0.2, respectively.

Interestingly, patients with pulmonary infections had significantly higher PON1 than those with nonpulmonary ones (respectively, 45 U (38.5-51.5) vs. 35.3 U (28-43), *p* = 0.049), but this association lost statistical significance when age-adjusted (*p* = 0.061).

There were no differences (*p* = 0.997) in PON1 activity in patients stratified by an invading pathogen with 39.3 U (21.5-57) in Gram-negative, 41.7 U (33-50.5) in Gram-positive, 38.2 U (29-47) in mixed MODS, and 39.6 U (29.5-50) in MODS with an unknown pathogen.

### 3.4. Independent Predictors of Admission PON1

Variables significantly associated with PON1 activity in univariate analysis, that is, the clinical type (with septic shock coded as 1), site of infection (abdomen against others), CVI, KF, and age, were entered into multivariate analysis to discern variables independently associated with the enzyme. The stepwise method of multiple regression was used, and the regression model was built explaining 50% of variability in PON1 activity with CVI (*r*_partial_ = −0.41, *p* = 0.019), abdominal MODS (*r*_partial_ = −0.46, *p* = 0.008), and age (*r*_partial_ = −0.39, *p* = 0.026) as explanatory variables. The effect of the clinical type and KF lost significance when coexamined with other variables.

### 3.5. PON1 during the Course of Observation

**Figure 4:**
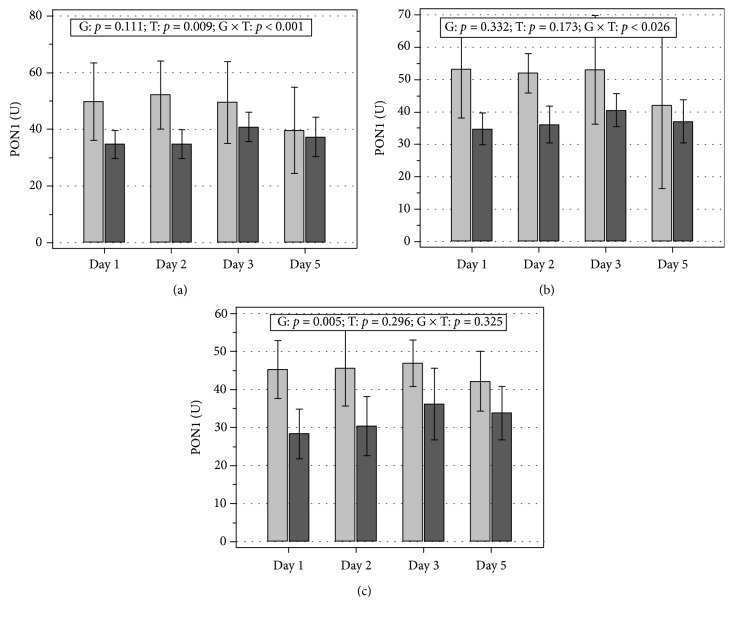
Time course of PON1 during a 5-day follow-up. Effect of the clinical type of sepsis (a), the presence of cardiovascular insufficiency (b), and the abdomen as an infection site (c). CVI: cardiovascular insufficiency; G: significance of a difference between groups; T: significance of a difference between time points; G×T: significance of interaction between the factor and time.

The clinical type of MODS (*p* = 0.001) and CVI (*p* = 0.042) affected the PON1 time course. There were significant day-to-day differences in the enzyme activity in patients with severe sepsis (*p* < 0.001) and in those without CVI (*p* < 0.001). In turn, PON1 activities in patients with septic shock or CVI remained stably low (Figure 4). Consequently, the differences between septic shock vs. severe sepsis and CVI vs. non-CVI observed at admission and at the 2nd day became insignificant on the 3rd day (respectively, *p* = 0.125 and *p* = 0.056). Similarly, the activity of PON1 in MODS patients with sepsis other than abdominal tended to increase whereas this in patients with abdominal sepsis decreased, so the difference in PON1 observed at admission lost its significance already on the 2nd day (*p* = 0.086).

### 3.6. PON1 and Inflammatory Response

We examined the correlation between PON1 and CRP, IL-6, PCT, LBP, LPS, WBC, PLT, and patients' temperature on the 1st, 2nd, 3rd, and 5th days. PON1 is negatively correlated with IL-6, which is significant on the 2nd day (*r* = −0.38, *p* = 0.029). Exclusively in septic shock patients, PON1 correlated negatively with LBP (*r* = −0.42, *p* = 0.036) and positively with LPS (*r* = 0.43, *p* = 0.031) and with patients' temperature, both on the 1st (*r* = 0.47, *p* = 0.018) and on the 2nd (*r* = 0.59, *p* = 0.002) days. Of these, LPS (*b* = 0.15, *p* = 0.034) and temperature (*b* = 4.74, *p* = 0.020) were independently associated with PON1 in septic shock patients, explaining 36% in data variability, both in unadjusted and age-adjusted models, regardless of the regression method applied.

### 3.7. Other PON1 Measures Derived from a 5-Day Follow-Up: PON1 Rates (ΔPON1) and Mean PON1 Activity (mPON1)

PON1 rates were not significantly associated with the clinical type (severe sepsis vs. septic shock), patients' outcome (survivors vs. nonsurvivors), infection site, acute organ dysfunction, and invading pathogen. Neither did ΔPON1 correlate with changes in any other biochemical or clinical parameter measured except for ΔPON1 paralleling changes in temperature (*r* = 0.61, *p* = 0.036) and WBC (*r* = 0.57, *p* = 0.052) but exclusively in severe sepsis patients. However, ΔPON1 correlated with the number of failed organs (*ρ* = -0.36, *p* = 0.037).

**Figure 5:**
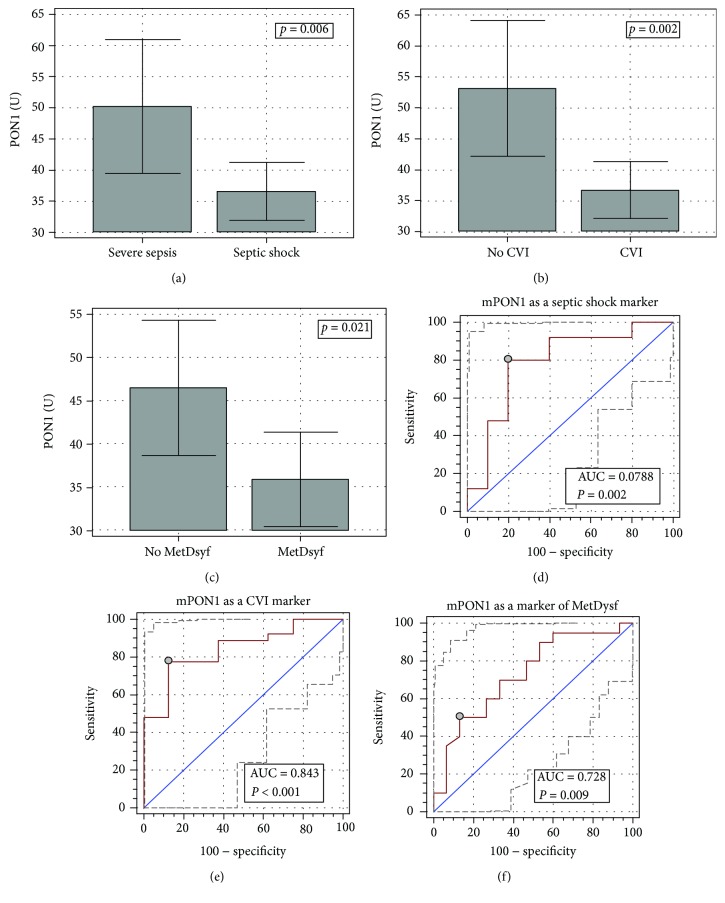
The association of mPON1 with the clinical type of MODS (a), cardiovascular insufficiency (b), and metabolic dysfunction (c) and its diagnostic power as a marker of septic shock (d), cardiovascular insufficiency (e), and metabolic dysfunction (f). Data on panels (a–c) presented as means with 95% CI and analyzed using the *t*-test for independent samples. Data on panels (d-e) presented as ROC curves with 95% CI. The diagonal line represents the chance marker with no discriminative power (AUC = 0.5), and a grey dot represents an optimal cut-off value. AUC: area under the receiver operating characteristic (ROC) curve; CVI: cardiovascular insufficiency; MetDysf: metabolic dysfunction.

The mPON1 was significantly lower in septic shock patients than in severe sepsis patients (Figure 5(a); *p* = 0.020 following age adjustment), in patients with vs. without CVI (Figure 5(b); *p* = 0.015 following age adjustment), and in patients with vs. without acute metabolic dysfunction (Figure 5(c); *p* = 0.004 following age adjustment). The mPON1 tended to be lower in patients with abdominal vs. nonabdominal infection (35.1 U (26.3-43.8) vs. 44.1 U (38.4-49.8) and *p* = 0.062 and *p* = 0.076, respectively, following age adjustment).

Of the age, clinical type, CVI, and metabolic dysfunction, CVI was independently associated with mPON1 activity (stepwise method of logistic regression). 26% of mPON1 variability was explained with CVI (*r*_partial_ = −0.51, *p* = 0.002). Exclusively in septic shock patients, mean PON1 positively correlated with mean temperature (*r* = 0.53, *p* = 0.007).

The mPON1 was a fair indicator of septic shock, characterized by 79% accuracy and, at a cut-off of ≤42.1 U, by 80% sensitivity and specificity and by LR+ of 4.0 and LR- of 0.25. Similarly, it was a fair marker of metabolic dysfunction, characterized by 73% accuracy and, at a cut-off of ≤34.4 U, by 50% sensitivity and 86.7% specificity and by LR+ of 3.8 and LR- of 0.58. The mPON1 was, in turn, a good marker of CVI with 84% accuracy and 77.8% sensitivity and 97.5% specificity accompanying at a cut-off of ≤42.1 U (Figures 5(d)–5(f)). Corresponding LR+ and LR- were 6.2 and 0.25, respectively.

## 4. Discussion

As a multifunctional enzyme, which anti-inflammatory, antioxidant, and quorum-sensing activities are potentially relevant for sepsis development, PON1 arouses special interest. All published studies on PON1 unambiguously and unanimously indicate its diminished activity in sepsis as compared to both healthy individuals [16, 18–20] and ICU patients without sepsis [17] as well as the enzyme restoration during recovery [16]. Moreover, a drop in PON1 activity could be observed regardless whether its paraoxonase [16, 19, 20], diazoxonase [19], or arylesterase [16–18] activities are assessed. Also in our cohort, arylesterase activity of PON1 was significantly lower in septic patients with MODS and displayed excellent accuracy in discerning MODS patients. So far, PON1 suitability as a diagnostic marker in sepsis has been evaluated only for enzyme paraoxonase and diazoxonase activities and yielded similar results with excellent and good accuracy, respectively [19]. However, PON1 arylesterase activity seems to be better suited for potential PON1 application in clinical practice as, unlike paraoxonase and diazoxonase activities, it is not affected by common PON1 polymorphisms and is thus representative of enzyme concentration [31]. Moreover, contrary to paraoxon and diazoxon, phenyl acetate, used as a substrate for determining arylesterase activity of PON1, is not toxic. Nevertheless, while indicative of strength of association, the excellence of PON1 in discriminating sepsis patients from healthy individuals is unlikely to find an application in clinical practice. In turn, a need for diagnostic biomarkers in sepsis discriminating between sepsis and noninfectious critical illness that would limit unnecessary use of antibiotics has been declared [33]. PON1 determination holds potential since Draganov et al. [17] found PON1 to significantly drop in sepsis, also as compared to critically ill patients without infection. However, diagnostic accuracy of the enzyme has not been evaluated, and therefore, PON1 suitability in this capacity has yet to be determined.

All previous studies on PON1 in ICU patients have been focused on the possible link between the enzyme and patients' survival and potential usefulness of PON1 as a prognostic marker [17–20]. PON1 activity has been found to decrease more pronouncedly in sepsis nonsurvivors [18, 20], and a drop in its associated activity reduces the overall survival [18] or higher risk of death [19]. There was no significant difference in PON1 between survivors and nonsurvivors in our patients. The discrepancy may result from the fact that our cohort of septic patients included only patients with the severest condition as reflected by higher APACHE II and SOFA scores and hsCRP concentrations than those reported by other authors [18, 19], and therefore, the range of PON1 activity in our study was likely to be narrower. Indeed, although not correlated with either of the clinical scores, we showed PON1 activity to be significantly more reduced in septic shock than severe sepsis, thus demonstrating its association with condition severity. Also, some authors found PON1 activity on admission to be insignificantly lower in nonsurvivors [17, 19] and only to drop substantially later in a follow-up [17], the observation we were unable to confirm due to too short observation time. Moreover, İnal et al. [20] found PON1 importance as a survival predictor negligible as compared to IL-6 or TNF*α*.

Nevertheless, we found PON1 activity to be associated with predictors of both the short-term and long-term outcomes. Short-time survival in sepsis is strongly associated with its initial severity [2], as already mentioned; admission as well as mean PON1 was substantially lower in septic shock. In turn, the increasing age and chronic illness remain important factors for predicting long-term survival [2]. Consistently, the age was negatively correlated with PON1 in our patients and was an independent predictor of enzyme activity on admission.

However, our primary objective was to assess, to the best of our knowledge for the first time, PON1 association with the infection site, pathogen, and the failure of specific organs. We found PON1 activity to drop significantly more pronouncedly in abdominal MODS what might be associated with the intestine to be additional to the liver site of PON1 synthesis [7]. Correspondingly, diminished PON1 activity in inflammatory bowel conditions has previously been reported by our group [13]. The gut has previously been considered “the ‘motor' of multiple organ failure” because of its ability to amplify systemic inflammatory response syndrome as its increased permeability facilitates entering of bacterial toxins and their propagation to distant organs *via* the lymphatic system [34]. It is hence possible that diminished PON1 in abdominal sepsis, through its abolished quorum-sensing and quorum-detoxifying activities, contributes to MODS.

Patients' survival can be improved by timely prediction of the disease course and the occurrence of complications, and as such, biomarkers allowing for identification of patients at a high risk of complications' development are looked after [35]. In order to evaluate PON1 suitability in this capacity, we assessed PON1 power in differentiating MODS patients with septic shock and severe sepsis and found it to be fair. PON1 accuracy would probably be even higher when tested on the population of ICU patients including those without MODS. We also sought PON1 association with the failure of specific organs. As circulating PON1 is derived mainly from the liver, we speculated that the enzyme might drop more noticeably in response to its failure. However, of all the evaluated organs/systems, a significantly more prominent drop in PON1 activity accompanied only CVI and PON1 was characterized by good accuracy as a CVI marker. This finding agrees well with known involvement of PON1 in cardiovascular diseases. Its diminished activity, particularly the arylesterase one, has been associated with an increased risk for major adverse cardiac events, such as death, myocardial infarction, and stroke. Moreover, PON1 provided the prognostic value independently from traditional risk factors, creatinine clearance, or elevated CRP among others [36]. The latter suggests that PON1 contributes apart from its anti-inflammatory activity. Our finding on the poor correlation between PON1 and inflammatory indices, that is, only a weak correlation with IL-6 on the 2nd day, corroborated by others [19], substantiates the notion. Accordingly, some authors [17, 19] found PON1 in ICU patients to be associated rather with the markers of oxidative imbalance. Also, corroborating our observation on PON1 close relation with CVI, Bojic et al. [19] reported PON1 activity to be inversely correlated with lactate concentrations, a marker of systemic hypoxia [37], and to be lower in patients with mechanical ventilation. Counterintuitively, PON1 was less diminished in septic patients with KF. Still, corroborating findings of other authors [38], their enzyme activity was significantly lower than in health. As PON1 activity is easily modified [39], it is likely that the retention of one or more metabolites or drugs in blood, caused by the impaired rate of glomerular filtration, may positively affect the enzyme. Consequently, KF patients would have relatively higher activity of the enzyme. The likely candidate is uric acid, a potent serum antioxidant [40]. PON1 activity is negatively affected by oxidative stress [38], and it is possible that higher uric acid concentrations observed in patients with renal dysfunction [41] may protect, to some extent, PON1 from oxidative stress.

It has been repeatedly suggested that following changes in biomarker levels throughout the disease course owns a potential greater than their measuring at admission [23, 25, 42]. The underlying rationale is that clinical improvement depends on the attenuation of inflammation, which is a gradual process, while admission levels of inflammatory mediators are subjected to interindividual variation and additionally affected by variability in the disease time points at which patients are admitted to the intensive care units. As already mentioned, changes in PON1 activity during a follow-up, but not its admission activity, have been associated with patients' survival [17]. We, in turn, in addition to differences in admission PON1 activity, found the enzyme dynamics to be distinct in association with the CVI and clinical type. PON1 activity was constantly low throughout the whole course of observation in patients with CVI and septic shock but steadily decreased in patients without CVI and with severe sepsis. To express time-course changes in PON1 in our patients, we calculated its mean activities throughout the follow-up as well as devised simple rates (ΔPON1). Of these, mean PON1 activities reflected associations observed for admission PON1, and, additionally, an association with metabolic dysfunction has been found. As a marker, mPON1 displayed slightly better accuracy than admission PON1. Simple rates, in turn, were poorly related to patients/sepsis characteristics. However, unlike absolute PON1 on any day of a follow-up or mean PON1, ΔPON1 correlated with a number of failed organs.

## 5. Conclusions

PON1 activity is significantly reduced in MODS, more so in older patients and in patients with CVI and the abdomen as a site of infection. Patients with septic shock, CVI, and abdominal MODS had distinctly different dynamics of PON1 during a 5-day follow-up. A drop in the enzyme activity was an excellent marker of MODS, a good marker of CVI, and a fair one of septic shock and abdominal sepsis. Additionally, mean PON1 during a follow-up was a fair marker of metabolic dysfunction in patients with MODS.

## Figures and Tables

**Table 1:** Characteristics of critically ill patients with MODS.

	Severe sepsis (*n* = 10)	Septic shock (*n* = 25)	*p* value
Gender, F/M	6/4	12/13	0.527^F^
Age (yrs. (range))	50.5 (18-72)	55 (21-91)	0.189^M^
Creatinine (mg%)	1.71 (0.92-3.19)	1.59 (1.13-2.22)	0.814^t^
Bilirubin (mg%)	0.98 (0.68-1.43)	1.03 (0.78-1.37)	0.845^t^
PLT (×10^3^/mm^3^)	99 (65-134)	155 (99-212)	0.083^W^
WBC (×10^3^/mm^3^)	12.1 (5.4-18.8)	16.9 (10.2-23.6)	0.374^t^
CRP (mg/L)	220.6 (105-336)	269.4 (201-338)	0.430^t^
PCT (*μ*g/L)	2.55 (0.69-9.41)	6.96 (3.14-15.46)	0.168^t^
IL-6 (ng/L)	73.7 (31.2-174.1)	630 (259-1536)	0.006^t^
LBP (mg/L)	44.2 (26.8-54.3)	47.7 (42-51.4)	0.361^M^
ICU stay (days (range))	7 (4-125)	13 (6-180)	0.018^M^
Survivors/nonsurvivors (*n*)	8/2	10/15	0.035^F^
APACHE II (range)	20 (14-31)	24 (18-28)	0.622^M^
SOFA (range)	6 (4.5-10)	10 (7-12)	0.092^M^
Infection site (*n*)			0.596^*χ*2^
Abdominal	2	7	
Pulmonary	5	13	
Blood	0	1	
Other	3	3	
Unknown	0	1	
Number of organ failures (*n*)			0.058^*χ*2^
2	4	3	
3	3	7	
4	2	9	
5	1	4	
6	0	2	
Organ/system dysfunction (*n* (%))			
Cardiovascular	2 (20)	25 (100)	<0.001^F^
Pulmonary	8 (80)	25 (100)	0.076^F^
Renal	6 (60)	12 (48)	0.711^F^
Hepatic	2 (20)	3 (12)	0.610^F^
Hematologic	6 (60)	14 (56)	1^F^
Metabolic	4 (40)	16 (64)	0.266^F^
CNS	2 (20)	0	0.076^F^
Pathogen (*n*)			0.243^*χ*2^
Gram-positive	2	8	
Gram-negative	1	6	
Mixed	1	5	
Unknown	6	6	

F, Fisher's exact test; M, Mann-Whitney U test; t, t-test for independent samples; W, Welch test; *χ*^2^, Ch-square test.

## Data Availability

The raw data used to support the findings of this study are available from the corresponding author upon reasonable request.
